# Toxicity Assessment of Mesoporous Silica Nanoparticles upon Intravenous Injection in Mice: Implications for Drug Delivery

**DOI:** 10.3390/pharmaceutics14050969

**Published:** 2022-04-30

**Authors:** William M. MacCuaig, Abhilash Samykutty, Jeremy Foote, Wenyi Luo, Alexander Filatenkov, Min Li, Courtney Houchen, William E. Grizzle, Lacey R. McNally

**Affiliations:** 1 Stephenson Cancer Center, University of Oklahoma, Oklahoma City, OK 73104, USA; wmaccuaig@ou.edu (W.M.M.); abhilash9samykutty21@gmail.com (A.S.); wenyi-luo@ouhsc.edu (W.L.); alexander-filatenkov@ouhsc.edu (A.F.); min-li@ouhsc.edu (M.L.); courtney-houchen@ouhsc.edu (C.H.); 2 Department of Biomedical Engineering, University of Oklahoma, Norman, OK 73109, USA; 3 Department of Microbiology, University of Alabama at Birmingham, Birmingham, AL 35294, USA; jbf130@uab.edu; 4 Department of Pathology, Oklahoma Health Science Center, Oklahoma City, OK 73104, USA; 5 Department of Medicine, Oklahoma Health Science Center, Oklahoma City, OK 73049, USA; 6 Department of Pathology, University of Alabama at Birmingham, Birmingham, AL 35294, USA; wgrizzle2@gmail.com; 7 Department of Surgery, Oklahoma Health Science Center, Oklahoma City, OK 73104, USA

**Keywords:** mesoporous silica nanoparticles, nanotoxicity, chitosan, poly (ethylene glycol)

## Abstract

Nanoparticles are popular tools utilized to selectively deliver drugs and contrast agents for identification and treatment of disease. To determine the usefulness and translational potential of mesoporous silica nanoparticles (MSNs), further evaluations of toxicity are required. MSNs are among the most utilized nano-delivery systems due to ease of synthesis, pore structure, and functionalization. This study aims to elucidate toxicity as a result of intravenous injection of 25 nm MSNs coated with chitosan (C) or polyethylene glycol (PEG) in mice. Following acute and chronic injections, blood was evaluated for standard blood chemistry and complete blood count analyses. Blood chemistry results primarily indicated that no abnormalities were present following acute or chronic injections of MSNs, or C/PEG-coated MSNs. After four weekly administered treatments, vital organs showed minor exacerbation of pre-existing lesions in the 35KPEG-MSN and moderate exacerbation of pre-existing lesions in uncoated MSN and 2KPEG-MSN treatment groups. In contrast, C-MSN treatment groups had minimal changes compared to controls. This study suggests 25 nm MSNs coated with chitosan should elicit minimal toxicity when administered as either single or multiple intravenous injections, but MSNs coated with PEG, especially 2KPEG may exacerbate pre-existing vascular conditions. Further studies should evaluate varying sizes and types of nanoparticles to provide a better overall understanding on the relation between nanoparticles and in vivo toxicity.

## 1. Introduction

In disease treatment, nanomedicine is a potential solution that provides theranostic capabilities for a variety of applications. Of the wide diversity of nanomaterial formulations, mesoporous silica nanoparticles (MSNs) have emerged as a popular option due to the ability to facilitate specific cargo delivery for imaging, and/or therapeutics [1]. The increase in popularity of MSNs as nano-delivery vehicles owes to uniformity in synthesis, tunability, and facile functionalization [2,3]. MSNs can be synthesized in a uniform size range and the mesoporous structure can encapsulate a due for treatment or medical imaging using clinically relevant modalities including MRI or fluorescence [4,5]. Surface modifications of MSNs permit targeting to disease-specific features within the body, such as unique extracellular receptors [6,7,8]. With such advantages, MSNs are intriguing for potential translation to a clinical setting.

Despite the potential for theranostic application of MSNs, concerns of toxicity have been partially responsible for impeded clinical translation. Historically, toxicity as a result of silica was associated with utilization of micro-scale particles, resulting in the development of autoimmune diseases [9]. Studies using silica nanoparticles have resulted in fibrosis and liver/kidney injury due to rapid renal clearance or sequestration to the liver [10,11]. In contrast, several in vivo studies indicate that silica nanoparticles are not toxic [12,13]. Inconsistent results in the evaluation of MSNs, and many types of nanoparticles, often occur due to multiple confounding factors in the nanoparticle system. That is, the nanoparticle type, cargo, functionalization, conjugation linkers, etc., all contribute to the toxicity response, or lack thereof. With the increasing popularity and opportunity for clinical translation, head-to-head comparisons of MSNs with commonly used functionalization are required.

As mentioned, evaluation of the toxicity of functionalized MSNs is crucial for translational studies. MSNs are rarely used without being functionalized via external coating; cargo would rapidly exit from the porous silica into the surrounding environment without inclusion of an external coating for MSNs. In addition, such coatings are often utilized to provide stealth in the body and/or specificity for application, thereof absence would greatly diminish efficacy [14,15,16]. While studies have observed that the use of an external coating on MSNs can mitigate toxicity and fibrosis [17], there are few head-to-head comparisons that carry clinical implications.

Polyethylene glycol (PEG) is a popular biocompatible polymer. When functionalized to nanoparticles such as MSNs, PEG adds stealth/targeting to permit nanoparticles to escape phagocytosis, leading to longer circulation in the bloodstream [18]. PEG can degrade in vivo, permitting cargo release, while maintaining minimal non-specific interactions [19]. However, PEG has been found to decrease uptake and interactions with target cells through too much of “stealth effect”, leading to decreased theranostic efficacy [20,21]. PEGylated nanomaterials have also been associated with renal toxicity and anaphylactic shock in vivo [22,23]. Chitosan is another commonly used biomaterial and nanoparticle coating in vivo. Chitosan is a deacylated form of naturally occurring polysaccharide chitin, and often is used as a nanomaterial due to pH-responsivity in biological relevant levels [24,25,26,27]. Protonation of an amine results in charge-based molecular swelling, allowing cargo release specifically in acidic environments, including malignancies [28]. Chitosan also provides a “stealth effect” like PEG, since chitosan is naturally occurring, although positive surface potential often leads to non-specific interactions in the body [27]. While studies have successfully used chitosan as a non-toxic nanomaterial [27,29], some studies report toxicity as a result of agglomeration [30,31], warranting further investigation. PEG and chitosan are proper examples of nanomaterials that should be evaluated in head-to-head toxicity studies as MSN external coatings.

This study sought to isolate, assess, and compare the relative toxicity elicited as a result of MSNs coating. Such a study to isolate the base silica nanoparticle and external coating, without cargo and further functionalization, has thus far been absent from the literature, particularly when evaluating toxicity with both single and multiple injections. Most often, nanoparticle formulations are complex with numerous components preventing specific assessment of an individual component, i.e., coating, and overall leading to inconsistent toxicity results. Determination of the toxicity elicited by various aspects of a single nanoparticle system, e.g., nanoparticle base material, shape, size, conjugation, coating, drug, etc., is near impossible. To maintain clinical similarity to treatments such as chemotherapy, MSNs of each type would be intravenously injected once for acute assessment, and once a week for a month for chronic assessment. Following treatment with MSNs or coated MSNs, blood can be measured for complete blood count and blood chemistry, both in the short term, and longer term following multiple injections. Utilization of an immunocompetent mouse model provides key information relating to the toxicity as a result of MSN treatment. CD-1 female mice were the subject of this study due to extensive history of utilization for toxicology and pharmacology experimentation [32,33,34]. Large size, tail vein access, and low aggressiveness contribute to popularity of CD-1 mouse utilization in such studies. Following blood tests, mouse organs were harvested to evaluate if treatments caused toxicity through histopathology.

## 2. Materials and Methods

### 2.1. Materials

Reagents from Sigma Aldrich (St. Louis, MO, USA): CTAB (hexadecyltrimethylammonium bromide) (molecular biology, ≥99%, H6269), low molecular weight chitosan (448,869), 2K PEG (poly (ethylene glycol)) (MW: 2000, 84,797), 35K PEG (poly (ethylene glycol)) (MW: 35,000, 81,310), GPTMS (3-glycidyloxypropyl) trimethoxysilane (≥98%, 440,167), TMOS tetramethyl orthosilicate (≥99%, 341,436), benzoylated dialysis tubing (D7884), TEA triethanolamine (gas chromatography, ≥99.0%, 90,279). Other materials and reagents: Milli-Q water dispensed through Milli-Pore Sigma (Sigma-Aldrich, St. Louis, MO, USA); glacial acetic acid (certified ACS, ≥99.7%, A38C-212) (Waltham, MA, USA); ethyl alcohol (anhydrous, 200 proof) (Warner Graham Company, Cockeysville, MD, USA); Dulbecco’s modified Eagle medium (DMEM) (ThermoFisher Scientific, Waltham, MA, USA); fetal bovine serum (FBS) (R&D Systems, Minneapolis, MN, USA).

### 2.2. Nanoparticle Synthesis

**Figure 1:**
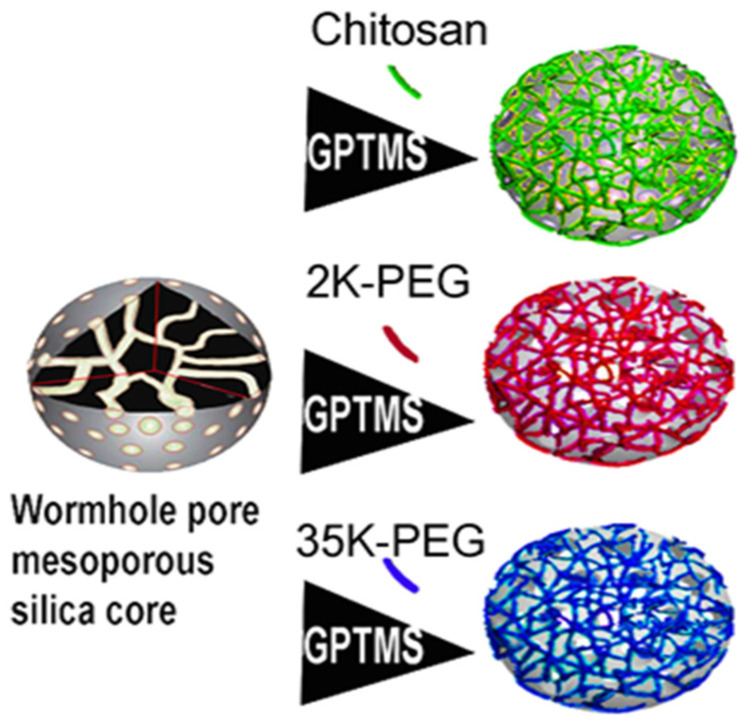
Schematic illustration of preparation of coated MSN preparation. Following synthesis of base MSNs, GPTMS linker is attached for subsequent chitosan, 2KPEG, or 35KPEG conjugation.

Mesoporous silica nanoparticles (MSNs) were prepared by modified Stober synthesis (Figure 1). A total of 2.00 g of CTAB was added to 240 mL of purified MQ water at 80 °C. After allowing the solution to reach 80 °C again, 0.42 g of TEA was added to the solution with moderate (400 rpm) stirring. Once heated to 80 °C, 11.0 mmol of TMOS was added and stirred on heat for 16 h. At completion, the reaction bottle was removed from heating/stirring and allowed to cool to room temperature. To remove the CTAB surfactant scaffold from the MSNs, a 1:1 (*v*/*v*) solution of 2 M glacial acetic acid and ethanol was used to clean the MSNs. About 50 mL of MSNs were added to 2000 MWCO dialysis tubing and placed into a 1 L solution of aforementioned acid–ethanol mix with a stir bar (100 rpm). External solution was replaced every 2 h for the first three cycles, and then every 12 h for the next five cycles. MSNs were then dialyzed similarly in MQ water for four cycles, each cycle lasting 12 h. MSNs were removed from dialysis bags and stored in a glass bottle.

### 2.3. Nanoparticle Characterization

A Malvern Zetasizer Nano ZS (Malvern Panalytical, Malvern, UK) was used to analyze MSN diameter, PDI, concentration, and zeta potential following synthesis, and throughout dialysis. Size parameter measurements were collected in triplicate using multi-angle dynamic light scattering (MADLS) at three angles (173°, 90°, 7°). Particle concentration was determined using MADLS with an input of 145 kcps for dispersant mean count rate. An Eppendorf Vacufuge 5301 was used to lyophilize MSNs at a rate of 0.2 mL/h. A TriStar analyzer was utilized to vacuum dry the powders at 120 °C overnight. A seven-point Brauner, Emmett, Teller (BET) isotherm and a 50-point adsorption/desorption isotherm were collected. Specific surface area and pore volume calculations were acquired through BET and Barrett–Joyner–Halenda (BJH) methodology [35].

### 2.4. Nanoparticle Functionalization

Dialyzed MSNs were diluted in ethanol (4:1) and ultrasonicated for 1 min at room temperature to ensure dispersion. MSNs were functionalized for subsequent attachment of polymer gatekeeper. Before coating, GPTMS (0.45 mmol, 0.1 mL) was added to 5 mL of 100% ethanol, and 20 mL of dialyzed MSN. The solution was gently stirred at room temperature for 3 h for grafting to MSNs. MSNs with silane linker were acidified to pH 3.5 via dropwise addition of 100 mM HCl for conjugation of chitosan. MSNs with silane linker were basified to pH 10 by addition of 100 mM NaOH for conjugation of PEG. 1% *w*/*v* chitosan, 2KPEG, or 35KPEG was added at a ratio of 6:5. Conjugation of chitosan/PEG occurred through epoxy ring opening of GPTMS. Solutions were shaken at 1000 rpms for 12 h at room temperature. Coated MSNs were isolated by centrifuging solutions at 15,000 rpm for 30 min at room temperature.

### 2.5. Functionalized Nanoparticle Characterization

Following synthesis of C-MSNs, 2KPEG-MSNs, and 35KPEG-MSNs, size, zeta potential, and PDI were reacquired to survey for differences from uncoated MSNs. MSNs, 0.5 mL of C-MSNs, 2KPEG-MSNs, and 35KPEG-MSNs (10^10^ particles/mL) were exposed to 10% FBS in standard DMEM cell growth media (1.5 mL) to simulate protein corona formation. After 24 h, each MSN group was revaluated using DLS for differences in size and zeta potential as a result of protein corona formation. Prior to coating conjugation, fluorescence contrast agent IR780 was encapsulated within C-MSNs, 2KPEG-MSNs, and 35KPEG-MSNs to observe mechanism of agent release in hypoxia and biological conditions (Supplementary Figure S1). IR780-loaded samples were pH adjusted to 6.6 or 7.4 diluted to an optical density of 1 OD/mL (V-730 UV-Vis Spectrophotometer, JASCO, Easton, PA, USA). Samples were shaken moderately until repeated measures at distinct time points. Absorbance of the supernatant was measured to determine release contrast agent.

### 2.6. In Vivo Nanoparticle Toxicity

Female CD1 immunocompetent mice aged 4 weeks were used in strict adherence to the University of Oklahoma Institutional Animal Care and Use Committee approved protocol. One week following receipt from Charles River Laboratories (Wilmington, NC, USA), mice were randomly divided into the following five groups:

1. Controls (C) n = 3.
2. Mesoporous silica nanoparticles with no coating (MSNs) n = 6.
3. Mesoporous silica nanoparticles coated with chitosan (C-MSNs) n = 6.
4. Mesoporous silica nanoparticles coated with 2 kDa polypropylene glycol (2KPEG-MSNs) n = 6.
5. Mesoporous silica nanoparticles coated with 35 kDa polypropylene glycol (35KPEG-MSNs) n = 6.

**Figure 2:**
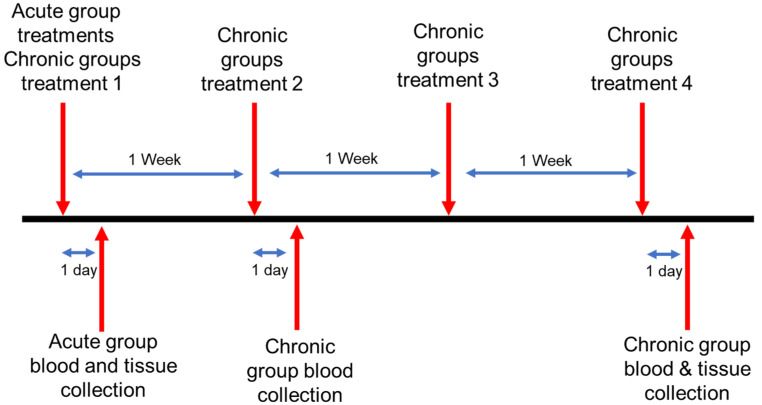
Timing schematic of in vivo toxicity studies. Acute and chronic group treatments began concurrently. Blood collections were held 24 h following select treatments. Tissue collections were held 24 h following the final treatment for respective treatment groups.

Except for controls, which received no injections, each animal received an intravenous tail injection of 10^10^ particles in 100 μL of treatments as noted in groups 2–5. Two points in time were studied; acute (24 h post-injection) and chronic (1 injection per week for 4 weeks) (Figure 2). The control animal samples were collected at the same time point as the chronic samples. About 24 h following treatment, blood samples were taken via retroorbital withdrawal from acute mice and analyzed by complete blood count (CBC) and blood chemistry (BC) tests. Chronic timepoint blood collection occurred after treatments 2 and 4, to minimize animal stress through overly frequent blood collection. Blood samples were analyzed using CBC and BC as well. Following completion of treatment, mice were euthanized via carbon dioxide overdose and cervical dislocation. Organs of interest (liver, spleen, kidney, pancreas, heart, and lung) were rapidly extracted from the euthanized mice and placed in neutral buffered formalin. After 16 h of fixation, tissues were processed to paraffin and embedded in paraffin blocks. Four micrometer tissue sections were cut from each tissue and stained with hematoxylin and eosin (H&E). Slides were analyzed blindly by both a board-certified veterinary pathologist (Foote) and a board certified diagnostic human pathologist with extensive experience in murine pathology (Grizzle, Luo, and Filatenkov).

### 2.7. Complete Blood Count and Chemistry Panels

Following retroorbital blood extraction from mice, complete blood counts were run using a Idexx Procyte DX Hematology Analyze (Idexx Laboratories, Westbrook, ME, USA) using edthylenediaminetetraacetic acid (EDTA) whole blood with 50 μL. Samples take 2 min to run. The Abaxis VetScan VS 2 (Zoetis, Parsippany, NJ, USA) was used for the blood chemistry panel with the Comprehensive Plus rotor. About 100 μL of lithium heparin (LH) whole blood was used to run samples in approximately 13 min.

### 2.8. Statistics

Statistical evaluation of the BC/CBC blood tests was performed using analysis of variance (ANOVA) and Tukey multiple comparison test with significance defined at *p* < 0.05. 

## 3. Results and Discussion

**Figure 3:**
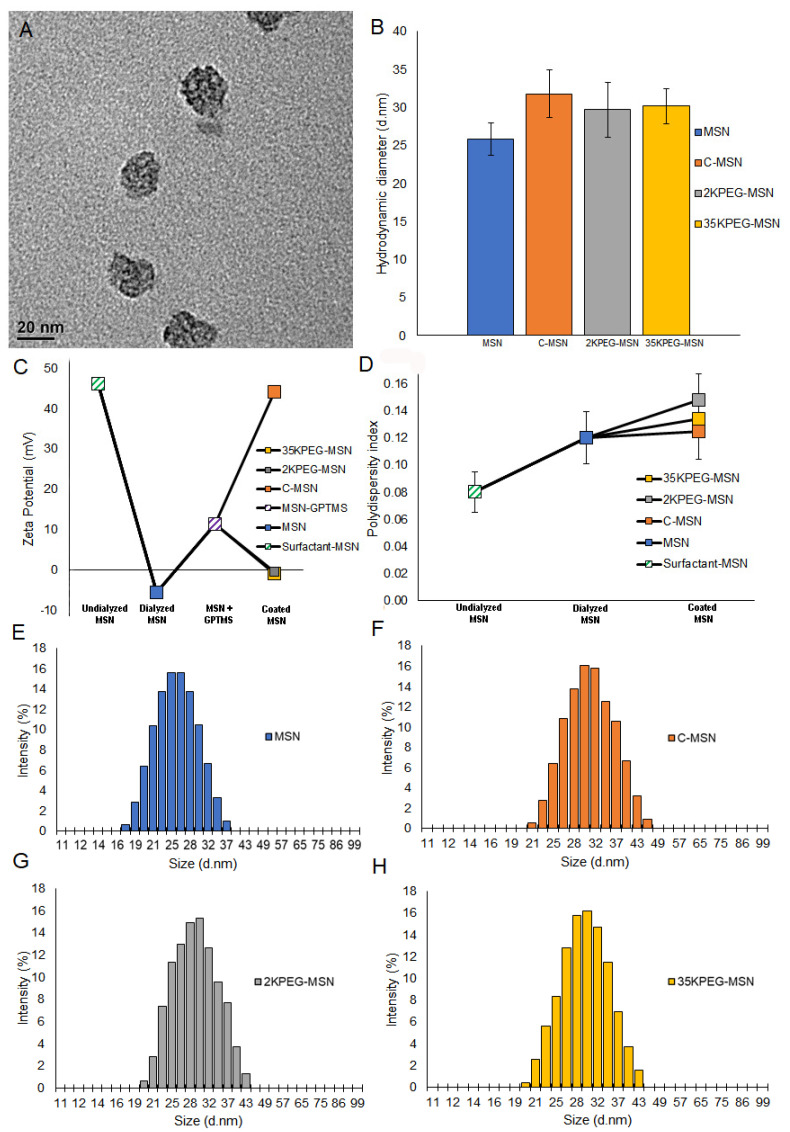
(**A**) Transmission electron microscopy image of synthesized MSNs. (**B**) Diameters of nanoparticles utilized in this study. Hydrodynamic diameters measured as 25.8 nm, 31.8 nm, 29.7 nm, and 30.1 nm for MSNs, C-MSNs, 2KPEG-MSNs, and 35KPEG-MSNs, respectively. (**C**) Zeta potential measurements of surfactant-MSNs, MSNs, C-MSNs, 2KPEG-MSNs, and 35KPEG-MSNs to ensure proper conjugation. (**D**) Polydispersity index of samples throughout synthesis. (**E**) Full MSN size distribution as determined by DLS measurements. (**F**) Full C-MSN size distribution as determined by DLS measurements. (**G**) Full 2KPEG-MSN size distribution as determined by DLS measurements. (**H**) Full 35KPEG-MSN size distribution as determined by DLS measurements.

MSNs were prepared through modified Stober synthesis. An emulsion of surfactant scaffold was formed under high temperature. Silica precursor coats the scaffold to create uniform MSNs of approximately 25 nm, measured with dynamic light scattering (DLS). While many sized MSNs are utilized, 25 nm has been identified as an intriguing size; large enough to avoid rapid glomerular filtration, but small enough to avoid sequestration by Kupffer cells in the liver [36]. Following preparation, MSNs are dialyzed to remove surfactant, preventing the possibility for surfactant-based toxicity. Prior to conjugation of coatings, nitrogen adsorption/desorption isotherms were analyzed with BET/BJH methodology to estimate MSN pore volume of 0.23 cm^3^/g, and MSN specific surface area of 750 m. Chitosan solution (1% weight by volume) was prepared in acetic acid and water. Due to controversy in toxicity as a result of PEG, PEG solutions with different molecular weights were utilized. PEG2000 and PEG35000 were selected due to a tradeoff between rapid clearance and extended circulation time that is relevant to drug delivery [37]. About 2% weight by volume solutions were prepared in water for molecular weights of 2000 (PEG2000) and 35,000 (PEG35000). Following surfactant removal, MSNs were diluted in ethanol and functionalized using an organosilane-epoxy linker for external coating attachment. PEG2000, PEG35000, and chitosan solutions were added to the MSN solution for attachment of coating. Average diameters were as follows: MSNs-25.8 (±2.1) nm; C-MSNs-31.8 (±3.1) nm; 2KPEG-MSNs-29.7 (±3.6) nm; and 35KPEG-MSNs-30.1 (±2.3) nm (Figure 3A,B). Full size distributions are included (Figure 3E–H). All polydispersity indexes were around or below 0.15, indicating monodisperse samples (Figure 3D). After synthesis, surfactant surrounding MSNs result in zeta potential measurements of 46 mV, which is reduced to −5.6 mV after surfactant removal, and increased to 11.3 mV following GPTMS addition (Figure 3C). Conjugation of chitosan was confirmed through increased zeta potential (44.1 mV) due to amine functional groups in physiological pH (7.4) [27,29]. Similarly, zeta potential for 2KPEG-MSNs (−0.6 mV) and 35KPEG-MSNs (−0.9 mV) increased slightly due to coating of silanol groups and decreasing external hydroxyl groups on MSNs, but not as dramatically as C-MSNs due to lack of amine functionality [38].

Addition of 10^5^ MSNs into a 1.5 mL solution of 10% FBS in cell growth media resulted in minor increases in coated MSN size and shifts in surface charge (Table 1). Small increases in size and slight alterations surface charge were observed in MSNs, 2KPEG-MSNs, and 35KPEG-MSNs. C-MSNs were observed to significantly increase in size by 17.1 nm to 48.9 nm following exposure to FBS. Chitosan has a relatively strong positive surface charge, which interacts with proteins in the FBS solution to form a protein corona. Formation of negatively charged proteins around C-MSNs results in a large drop in zeta potential, from +44.1 mV to 12.6 mV. These results suggest that C-MSNs will likely form a protein corona upon introduction into the body.

**Figure 4:**
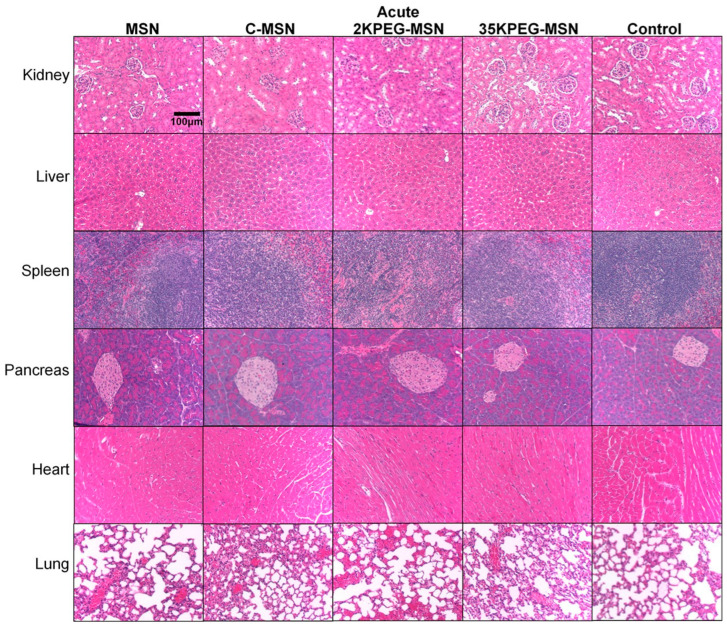
H&E-stained organs from acute treatment groups (MSNs, C-MSNs, 2KPEG-MSNs, 35KPEG-MSNs). Images were acquired at 10× magnification.

**Figure 5:**
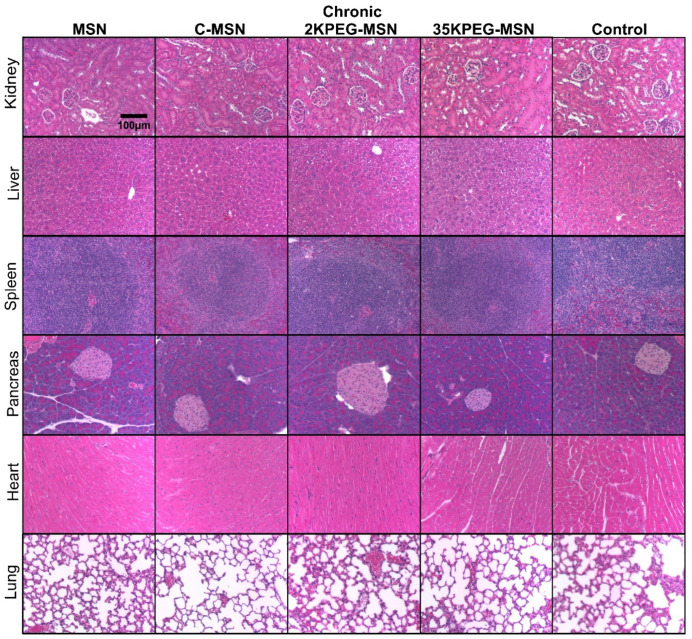
H&E-stained organs from chronic treatment groups (MSNs, C-MSNs, 2KPEG-MSNs, 35KPEG-MSNs). Images were acquired at 10× magnification.

CD-1 immunocompetent mice were utilized for testing in vivo toxicity. Mice were split into acute and chronic experiments, characterized by number of repeat treatments, toxicity evaluations, and time between treatments and measurements. Acute mice were intravenously injected with 10^10^ MSNs in 100 μL; chitosan-coated MSNs (C-MSNs), PEG2000-coated MSNs (2KPEG-MSNs), and PEG35000-coated MSNs (35KPEG-MSNs). About 24 h following treatment, blood samples were taken via retro-orbital withdrawal and analyzed by complete blood count (CBC) and blood chemistry (BC) tests (Table 2). In addition to short-term evaluations, toxicity concerns over time are of great importance and clinical relevance when considering multiple injections. To elucidate chronic toxicity, CD-1 immunocompetent mice were intravenously injected with treatments consistent with the short-term studies (MSNs, C-MSNs, 2KPEG-MSNs, 35KPEG-MSNs), once a week for a total of four treatments. Blood samples were taken via retro-orbital withdrawal 24 h following treatment 2 and 24 h following the final treatment. Blood was not collected after treatment 3 to avoid stress on mice due to frequent collection. Subsequent to final blood collection for both acute and chronic treatment groups, mice were euthanized via CO2 overdose and cervical dislocation. Organs were collected (liver, kidney, spleen, lung, pancreas, heart) for histopathologic evaluation (Figure 4 and Figure 5).

In general, the histopathology of acute and chronic time points for the C-MSN group was similar to controls and the mice had no areas of fibrosis in any organ or other signs of organ damage. The histological and blood chemistry findings from mice acutely or chronically injected with C-MSN were consistent with control mice. While it would be unlikely to utilize uncoated MSNs as either a drug or contrast agent delivery system, we found that uncoated MSN resulted in extensive right atrial right ventricle thrombus in one mouse.

In contrast, our study indicates that PEG may be a risk factor for increases in interstitial fibrin thrombi. Our findings show that 35KPEG-MSN had extensive interstitial fibrin thrombi in the alveoli, sinusoidal immature thrombi/hemorrhages, and had immature thrombi in the left atrium in one mouse. One mouse receiving 35KPEG-MSNs also had some hepatic necrosis. The lower molecular weight PEG 2KPEG-MSN had greater and more extensive immature thrombi in the pulmonary arteries and veins estimated to occupy 50%. There also was an extensive thrombus in the left ventricle. While the 35KPEG-MSN treatment group contained one animal with interstitial fibrin thrombi, all animals in the 2KPEG-MSN group were observed with extensive thrombi in 50% of vessels. This suggests that PEG may be a culprit in increased interstitial fibrin leading to immature thrombi and even mature thrombi as well as pulmonary aggregation. While lower molecular weight PEG has increased propensity for aggregation [39], this is the first study to report PEG, specifically 2KPEG, and also 35KPEG to a much lesser extent, as a risk factor of increased interstitial fibrin, immature thrombi, fully mature thrombi (in the 2KPEG-MSN group), and pulmonary aggregations following acute and chronic treatments of intravenously injected nanoparticles without extensive complexity, i.e., targeting peptides, drugs, etc.

Observed tissue changes varied among individual mice in each group and among groups. Of note, tissue changes did not affect organ function per blood analysis, animal weights, or animal behavior. There is concern for a interstitial fibrin and thrombi in the vasculature, alveoli, and large thrombi as all mice receiving 2KPEG-MSNs had similar vascular pathologies to a great extent and one animal out of six injected with or 35KPEG-MSNs to a much lesser extent. Tissues of mice receiving C-MSNs did not indicate a danger to the mice secondary to receiving these injections. Due to existing lesions in control mice, it is unlikely that these lesions would be induced de novo in cases receiving C- or 35KPEG-MSNs. At worst, the C-MSN or 35KPEG-MSN exacerbate existing conditions or because of these existing lesions may confuse comparisons. Because of preexisting vascular injury in arteries [40], our results may also suggest a more extensive healing process of injury for mice treated with MSNs and 2KPEG-MSNs compared to C-MSNs and 35KPEG-MSNs.

For the CD-1 mouse, the histopathology of mice is unusual. Specifically, hepatocytes have cytoplasm which are clear in the morning due to increased glycogen storage [41,42]. However, this may change in a few hours due to the fed-fasting cycle during the sleep cycle of the mouse, deeming it important to sacrifice the mice during the same temporal period [41,42]. This change has been described, but sometimes has led to incorrect interpretations that this change is instead a result of therapies. Control mice demonstrated no areas of fibrosis or acute inflammation. Two control mice had immature thrombi in heart, with one having immature thrombi and hemorrhage in the lung. Two of the control mice had multifocal areas of hepatic necrosis, and one was observed to have hepatic atrophy and areas of renal edema and hemorrhage. The control mice demonstrate three major potential pathologies, (1) areas of multifocal hepatocyte necrosis/atrophy; (2) multifocal areas of renal necrosis, edema, and hemorrhage; and (3) multifocal immature thrombi in the vasculature of the lungs, cardiac ventricles, atria, and pulmonary arteries. These lesions may be considered as secondary to existing pathologies as each was observed in control mice that received no intravenous injections throughout the experimentation period. Notably, thrombosis has been reported as an occasional change consistent with the aging of CD-1 mice [43,44,45]. Thrombi often occur secondary to spontaneous/induced vasculitis rather than a toxicologically induced legion, possibly explaining thrombi presence in control mice [46]. In this study, while no organ failure is observed, extensive areas of thrombi were present in the lungs of pre-disposed CD-1 mice. Following treatments, PEG-coated MSNs appeared to exacerbate such injuries, where 2KPEG-MSNs treatment groups had larger areas of extensive thrombosis, suggesting increased aggregation propensity of lower molecular weight PEG.

Blood tests revealed small, transient differences in 2KPEG-MSNs as compared to the other groups tested acutely. Specifically, phosphorous levels in the blood were elevated in the 2KPEG-MSN treatment group. High amounts of phosphorous in blood can be indicative of kidney disease, as kidneys are responsible for filtering and removing excess phosphorous from the blood [47]. Consistent with previous reports of kidney ailments, low molecular weight PEG may have negative impact on kidney function [22,23,48]. Specifically, PEG has shown to result in calcium phosphate crystal deposition in renal tubules [49]. The crystal deposition can lead to subsequent nephrocalcinosis and acute renal failure [49]. In addition, sodium levels in 2KPEG-MSN acute treatment groups were slightly elevated, suggesting possible dehydration, which could arise from many variables, including kidney dysfunction [50]. However, while transient differences were observed in the 2KPEG-MSNs acute treatment group, no differences were observed in the chronic treatment groups. No other biomarkers for kidney dysfunction (BUN, CRE) were significantly different in the 2KPEG-MSN groups, suggesting minor calcium phosphate crystal deposition [49]. Apart from these deviations in 2KPEG-MSN acute treatment groups, there were no other significant changes in acute or chronic levels as measured with blood chemistry and complete blood counts. PEG has a propensity to undergo cellular vacuolation [51,52] and these results suggest nanoparticles that utilize lower molecular weight PEG may be more at-risk for vacuolation.

## 4. Conclusions

Increased potential of nanomedicine in a clinical setting has raised significant concern in regards to toxicity. Nanoparticles can be difficult to evaluate for toxicity due to inconsistencies with administration routes and a wide variety of nanoparticle base materials, modifications of the surface of nanoparticles, gatekeepers, and even drugs. With typical multifactorial nanoparticles, the toxicity is often assessed with only a single injection, without proper isolation of each factor of the nanoparticle system such as external coatings, conjugated ligands, or encapsulated drugs. MSNs are a specialized set of nanomedicines that contain: (1) A wide range of size from 3 nm to >100 nm in diameter [53,54,55]; (2) adaptable gatekeeper mechanisms to allow for stimuli-responsive cargo release or stealthing [14,56,57]; and (3) may have additional disease specific-targeting [36,58,59]. However, there is controversy in assessment of MSN toxicity and accumulation due to the numerous and variable features within MSNs. For example, simultaneous determination of elicited toxicity as a result of the nanoparticle core, shape, size, conjugation, drug, etc., is nearly impossible.

This suggests that PEG can exacerbate pre-existing vascular conditions, but that PEG causes greater toxic impact than 35KPEG. This study evaluated MSN toxicity using uncoated MSNs, chitosan, low molecular weight PEG, and high molecular weight PEG administered intravenously in mice. Treatment groups were split into acute (1 injection) and chronic (1 injection per week over 4 weeks) for maintaining clinical relevance, i.e., single vs. multiple rounds of chemotherapy. The chitosan coating on MSNs (C-MSN) did not result in changes in histopathology of acute and chronic time points for the C-MSN group was similar to controls and the mice had no areas of fibrosis in any organ or other signs of organ damage. The histological and blood chemistry findings from mice acutely or chronically injected with C-MSN were consistent with control mice. In 100% mice receiving 2KPEG-MSNs, the mice had increased interstitial fibrin, immature thrombi, and even fully mature thrombi in up to 50% of the pulmonary arteries and veins. Additionally, one mouse receiving multiple injections 35KPEG-MSN also had increased interstitial fibrin and immature thrombi. Thus, a main finding of this study suggests chitosan coating was safe, but that PEG coating can exacerbate pre-existing vascular conditions with 2KPEG causing much greater toxic impact than 35KPEG.

Efficacious drug delivery has been observed in each of the chosen gatekeepers, chitosan [36], 2KPEG [60], and 35KPEG [61]. While 2KPEG shows rapid clearance from the body, 35KPEG benefits from a longer circulation time [37]. Histological evaluation of the tissue suggests exacerbation of pre-existing thrombotic lesions of mice treated with uncoated MSNs and PEG-coated MSNs. These results suggest that PEG may negatively impact and worsen mice that have pre-existing vascular risk. In addition, low molecular weight PEG results in increased exacerbation of vascular injury. Small differences were observed in the blood chemistry (PHOS, NA+) of acute low molecular weight PEG treatment group that indicated kidney dysfunction, but these results were not supported by other kidney-related biomarkers (BUN, CRE). Such differences may be a result of increased calcium phosphate depositions as a result of PEG coating [49], where lower molecular weight PEG may have a higher propensity for cellular vacuolation [51,52]. Additional investigations of many molecular weights of PEG and chitosan may provide more valuable information concerning how external coatings affect MSN toxicity. Further studies to accurately define the limits of toxicity depending on particle size, type, and application will improve translatability of MSNs and general nanomedicine from high potential to clinical translation.

## Figures and Tables

**Table 1:** Size and charge characteristics of MSNs, C-MSNs, 2KPEG-MSNs, and 35KPEG-MSNs following 24-h exposure to 10% FBS in cell growth media compared to as-synthesized MSNs.

FBS Treatment Characterizations	Diameter by DLS (nm)			Zeta Potential (mV)		
MSN Coating	As-Prepared	FBS-Treated	Change	As-Prepared	FBS-Treated	Change
MSN	25.8	28	+2.2	−5.59	−2.84	+2.75
C-MSN	31.8	48.9	+17.1	44.07	12.62	−31.45
2KPEG-MSN	29.7	29.9	+0.2	−0.58	−1.35	−0.77
35KPEG-MSN	30.1	30.3	+0.2	−0.91	−1.72	−0.81

**Table 2:** Blood chemistry values as reported from acute (A) and chronic (B) treatment groups. Complete blood count values as reported from acute (C) and chronic (D) treatment groups.

A	Blood Chemistry-Acute					B	Blood Chemistry-Chronic				
	Untreated	MSN	C-MSN	2KPEG-MSN	35KPEG-MSN		Untreated	MSN	C-MSN	2KPEG-MSN	35KPEG-MSN
ALB	4.5	4.37	4.57	4.57	4.4	ALB	4.53	4.53	4.67	4.63	4.57
ALP	110.3	88.7	104.3	118	110.3	ALP	82.3	95.7	91.7	92.7	80
ALT	38	34	49	51.3	28.3	ALT	38	30.7	36.7	23.7	35
AMY	1189	1163	1067	986	1083	AMY	1095	1066	1085	1174	1067
TBIL	0.33	0.4	0.4	0.4	0.3	TBIL	0.47	0.23	0.3	0.27	0.3
BUN	20	21.7	21.3	18.7	19.3	BUN	20.3	23.7	22.3	18.7	20.7
CA	11.8	11.3	11	11.8	11.1	CA	11.3	11	11.1	11.3	11.2
PHOS	8.17	7.7	7.97	9.6	7.7	PHOS	7.63	7.43	7.1	7.3	6.2
CRE	0.27	0.33	0.27	0.3	0.33	CRE	0.27	0.27	0.2	0.23	0.37
GLU	128.7	103.7	108.3	117.7	119.7	GLU	117.3	116.3	96.3	98.7	99
NA+	154.7	154.3	153.7	159	154	NA+	155.7	156	154.7	155.3	155.7
K+	6.9	6.43	6.87	6.67	5.97	K+	6.93	7	7.05	7.13	6.1
TP	6.03	5.8	6	6.07	5.9	TP	6.07	5.9	6.17	6.13	6.27
GLOB	1.53	1.43	1.43	1.5	1.53	GLOB	1.57	1.37	1.5	1.5	1.67
**C**	**Complete Blood Count-Acute**					**D**	**Complete Blood Count-Chronic**				
	Untreated	MSN	C-MSN	2KPEG-MSN	35KPEG-MSN		Untreated	MSN	C-MSN	2KPEG-MSN	35KPEG-MSN
RBC	10.2	10.5	10.3	10.3	10.5	RBC	10.4	10.2	11.4	10.7	10.8
HGB	16.6	17.2	16.4	16.2	16.7	HGB	16.7	15.8	17.1	16.4	16.3
HCT	56.9	58.4	55.4	55.1	58.3	HCT	56.8	54.5	57.6	56.2	56.1
MCV	56	55.5	54	53.6	55.4	MCV	54.6	53.4	50.6	52.6	51.9
MCH	16.3	16.3	16	15.7	15.8	MCH	16	15.5	15	15.3	15.1
MCHC	29.1	29.4	29.5	29.3	28.6	MCHC	29.3	29	29.7	29.2	29
RDW-SD	28.9	29.7	28.8	29	29.5	RDW-SD	30.1	30.1	29.1	29.3	29.7
RDW-CV	20.7	21.7	21.6	21.8	21.6	RDW-CV	22.1	22.6	24.1	22.9	23.4
RET	409	396	440	367	541	RET	282	380	562	477	564
RET%	4.05	3.78	4.32	3.57	5.18	RET%	2.69	3.77	4.96	4.4	5.22
PLT	904	776	979	878	1086	PLT	1173	1113	1548	1338	1360
PDW	9.1	9.37	8.5	10.1	9.13	PDW	9.53	7.67	9.83	8.13	7.73
MPV	8.1	7.97	8.23	8.47	8.3	MPV	8.43	8	9.3	8.23	8.17
P-LCR	12	10.9	9.4	12.9	10.1	P-LCR	12.5	6.5	10.7	7.1	6.7
PCT	0.73	0.62	0.8	0.74	0.92	PCT	0.99	0.9	1.41	1.1	1.1
WBC	4.44	5.67	6.7	6.7	6.02	WBC	4.41	5.32	7.74	3.81	6.09
NEUT	0.52	0.78	1.46	0.8	0.67	NEUT	0.46	0.79	0.85	0.45	0.79
LYMPH	3.79	4.72	5.03	5.74	5.19	LYMPH	3.81	4.37	3.77	3.23	5.1
MONO	0.02	0.03	0.01	0.03	0.04	MONO	0.01	0.03	0.02	0.03	0.05
EO	0.09	0.14	0.19	0.13	0.11	EO	0.12	0.13	0.1	0.09	0.15
BASO	0.01	0	0.01	0.01	0.01	BASO	0.01	0.01	0.01	0.01	0.01

Abbreviations: ALB—albumin, ALP—alkaline phosphatase, ALT—alanine transaminase, AMY—amylase, TBIL—total bilirubin, BUN—blood urea nitrogen, CA—calcium, PHOS—Phosphorus, CRE—creatinine, GLU—glucose, NA+—Sodium, K+—Potassium, TP—total protein, GLOB—globulins, RBC—red blood cells, HGB—hemoglobin, HCT—hematocrit, MCV—mean corpuscular volume, MCH—mean corpuscular hemoglobin, MCHC—mean corpuscular hemoglobin concentration, RDW—red cell distribution width, RET—reticulocyte, PLT—platelets, PDW—platelet distribution width, MPV—mean platelet volume, P-LCR—platelet/large cell ratio, PCT—procalcitonin, WBC—white blood cells, NEUT—neutrophils, LYMPH—lymphocytes, MONO—monocytes, EO—eosinophils, BASO—basophils.

## Data Availability

Not applicable.

## Supplementary Materials

The following supporting information can be downloaded at: https://www.mdpi.com/article/10.3390/pharmaceutics14050969/s1, Figure S1: Simulation of contrast agent IR780 exfiltration from MSN pores while coated with (A) Chitosan, (B) 2KPEG, or (C) 35KPEG. Treatments were added to 10% phosphate buffered saline at a biological pH level of 7.4 or a hypoxic pH level of 6.6.
