# Correction: MacCuaig et al. Toxicity Assessment of Mesoporous Silica Nanoparticles upon Intravenous Injection in Mice: Implications for Drug Delivery. *Pharmaceutics* 2022, *14*, 969

**DOI:** 10.3390/pharmaceutics18020174

**Published:** 2026-01-29

**Authors:** William M. MacCuaig, Abhilash Samykutty, Jeremy Foote, Wenyi Luo, Alexander Filatenkov, Min Li, Courtney Houchen, William E. Grizzle, Lacey R. McNally

**Affiliations:** 1 Stephenson Cancer Center, University of Oklahoma, Oklahoma City, OK 73104, USA; wmaccuaig@ou.edu (W.M.M.); abhilash9samykutty21@gmail.com (A.S.); wenyi-luo@ouhsc.edu (W.L.); alexander-filatenkov@ouhsc.edu (A.F.); min-li@ouhsc.edu (M.L.); courtney-houchen@ouhsc.edu (C.H.); 2 Department of Biomedical Engineering, University of Oklahoma, Norman, OK 73109, USA; 3 Department of Microbiology, University of Alabama at Birmingham, Birmingham, AL 35294, USA; jbf130@uab.edu; 4 Department of Pathology, Oklahoma Health Science Center, Oklahoma City, OK 73104, USA; 5 Department of Medicine, Oklahoma Health Science Center, Oklahoma City, OK 73049, USA; 6 Department of Pathology, University of Alabama at Birmingham, Birmingham, AL 35294, USA; wgrizzle2@gmail.com; 7 Department of Surgery, Oklahoma Health Science Center, Oklahoma City, OK 73104, USA

**Error in Figure**

In the original publication [1], there was a mistake in Figure 4 as published. The authors wish to correct the inadvertently duplicated histology image within the panel. This occurred as a version control error over the course of the revision process. The corrected Figure 4 appears below. The authors state that the scientific conclusions are unaffected. This correction was approved by the Academic Editor. The original publication has also been updated.

## Figures and Tables

**Figure 4:**
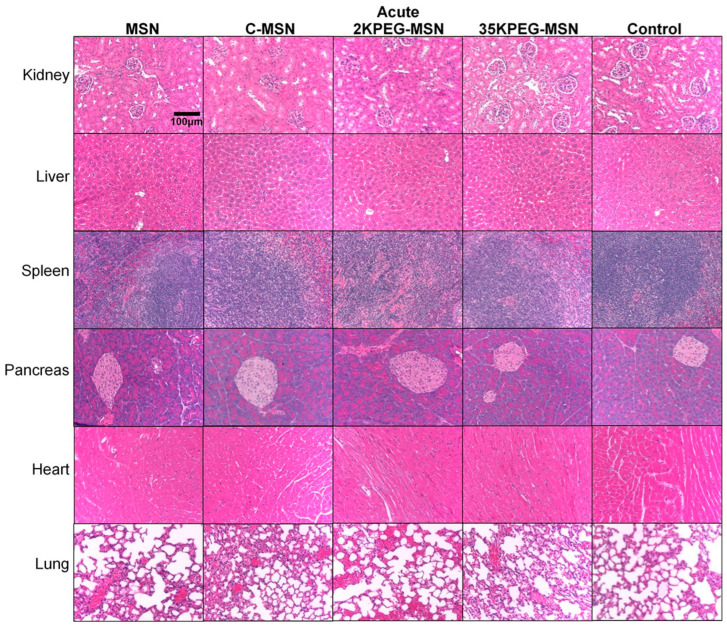
H&E-stained organs from acute treatment groups (MSNs, C-MSNs, 2KPEG-MSNs, 35KPEG-MSNs). Images were acquired at 10× magnification.
