# Supplementary material for: Toxicity Assessment of Mesoporous Silica Nanoparticles upon Intravenous Injection in Mice: Implications for Drug Delivery
Source: Pharmaceutics. 2022 Apr 30;14(5):969. doi: 10.3390/pharmaceutics14050969 (PMC9148138; doi:10.3390/pharmaceutics14050969)
Supplement: Supplementary file 1 [file pharmaceutics-14-00969-s001.zip › pharmaceutics-1672068-supplementary.pdf]

pharmaceutics

Supplementary Materials: Toxicity Assessment of Mesoporous Silica Nanoparticles upon Intravenous Injection in Mice: Implications for Drug Delivery

William M. MacCuaig, Abhilash Samykutty, Jeremy Foote, Wenyi Luo, Alexander Filatenkov, Min Li, Courtney Houchen, William E. Grizzle and Lacey R. McNally

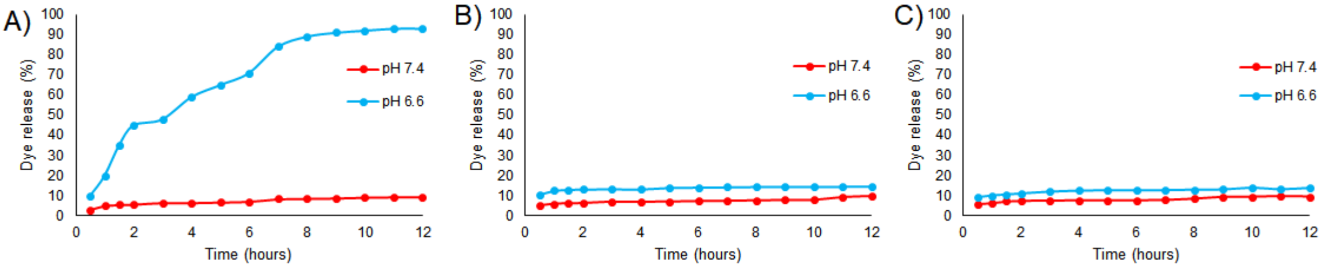

Figure S1. Simulation of contrast agent IR780 exfiltration from MSN pores while coated via (A) Chitosan, (B) 2KPEG, or (C) 35KPEG. Treatments were added to 10% phosphate buffered saline at a biological pH level of 7.4 or a hypoxic pH level of 6.6.
